# Impact of virtual reality on pain management in transrectal MRI-guided prostate biopsy

**DOI:** 10.3389/fpain.2023.1156463

**Published:** 2023-10-02

**Authors:** Emma Perenic, Emilie Grember, Sébastien Bassard, Nicolas Koutlidis

**Affiliations:** ^1^ Department of Urology, Chalon-sur-Saone Hospital, Chalon-sur-Saone, France; ^2^ Department of Urology, Dijon University Hospital, Dijon, France

**Keywords:** prostate biopsy, transrectal biopsy, image-guided biopsy, virtual reality, analgesia, pain distraction

## Abstract

**Background:**

The beneficial effect of virtual reality (VR) on pain management in the context of transrectal MRI-guided prostate biopsy is not well established. However, it remains unclear whether an adjunctive of VR also improves pain management. This study aimed to evaluate the impact of VR as adjunctive in pain management in transrectal MRI-guided prostate biopsy (PB).

**Methods:**

We retrospectively evaluated the pain intensity incidence in the 153 patients with PB indication (of which 102 were naïve of PB) who were admitted to our hospital since the acquisition of the Healthy Mind virtual reality headset on 19 January 2021.

**Results:**

Baseline characteristics of patients who received local anesthesia with 1% lidocaine periprostatic nerve block (PPNB) (Group SOC, *N* = 78) and patients who received VR associated with PPNB (Group VR, *N* = 75) were largely similar. One PB with general anesthesia was excluded. The mean pain score at day zero was respectively 3.4 (±2.5) and 2.9 (±2.3) for SOC and VR (*p* = 0.203). However, the mean pain score at day zero was significantly lower in naïve PB patients with VR [2.7 (±2.0)] than in naïve PB patients with SOC [3.8 (±2.5), *p* = 0.012] when patients were stratified in PB status. Similar results were found on day 3 for the analysis including naïve-PB patients with SOC vs. with VR [0.4 (±2.5) vs. 0.2 (±2.0); *p* = 0.023)].

**Conclusions:**

The pain intensity was significantly lower in naïve PB patients with VR than in naïve PB patients with SOC. There were no side effects from VR and tolerability was excellent.

## 1. Introduction

Prostate cancer ranks first among cancers in men, with 50,430 new cases estimated in 2015 in metropolitan France. Prostate cancer ranks third in cancer deaths in men, with 8,512 estimated deaths in 2015 (1, 2).

Despite the screening of cancer with the dosage of PSA, which consists of measuring the concentration in the blood of a protein synthesized by the prostate, the diagnosis is based on histological analysis of the prostate gland through biopsies. Prostate biopsies are performed either trans-rectally (the needle is inserted through the rectum to the prostate) or trans-perineally (the needle is inserted through the skin between the bursa and the anus to the prostate). It is a painful examination that requires local anesthetic and the preparation time can lead to anxiety in the patient.

Over the past decades, virtual reality (VR) has been proposed as a new way to manage pain in a non-pharmacological way. By diverting attention away from the symptoms and immersing the participant in a virtual environment, VR can profoundly alter pain perception (3). Several studies show its efficacy in managing pain during labor (4), burn dressing changes (5), periodontal scaling and root planing procedures (6), and for children undergoing medical procedures (7). Several methods of analgesia have been proposed (8–11), including periprostatic basal (12) or apical nerve block (13), topical anesthesia with lidocaine (14), nitrous oxide-oxygen inhalation (15), diclofenac suppository (16), or sedation (17). The national committee guidelines ccAFU currently recommend the periprostatic nerve block (PPNB) with or without topical gel instillation (18).

The purpose of this prospective study was to compare the efficacy and tolerability of virtual reality associated with periprostatic nerve block and periprostatic nerve block alone in patients undergoing transrectal MRI-guided biopsy.

## 2. Materials and methods

### 2.1. Study population

**Figure 1:**
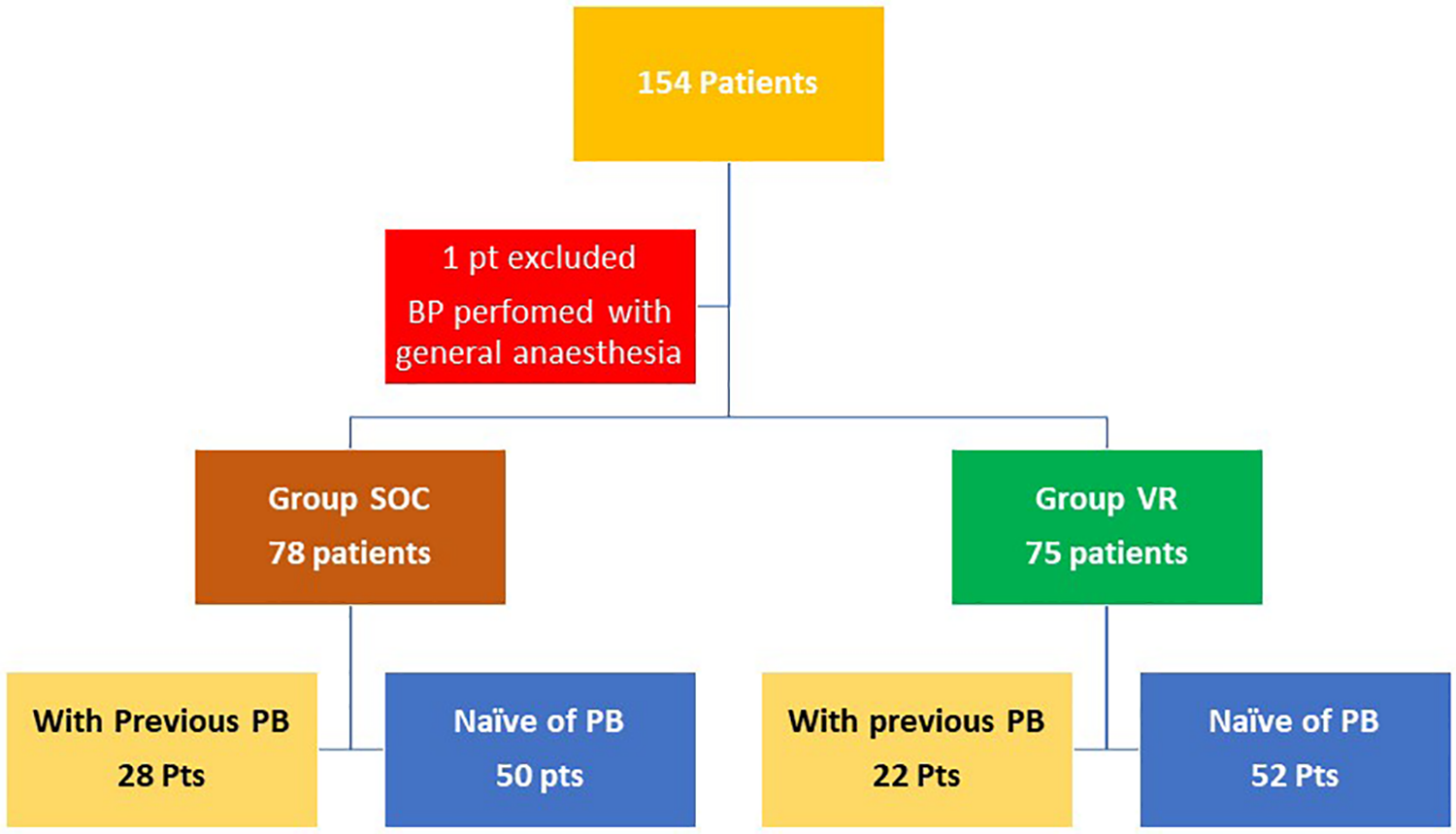
Flow-chart.

The department database is a prospective cohort initiated in the acquisition of a virtual reality headset on January 2019 that records all patients admitted for care in the Department of Urology, Chalon-sur-Saone Hospital (France). We included in our analysis all patients enrolled from 2019 to January 2021 meeting the following criteria: PB performed in the department. Exclusion criteria included the presence of general anesthesia or lack of a minimum follow-up of 3 days (Day 0 and Day 3) (Figure 1).

The study was conducted in accordance with the ethical guidelines of the Declaration of Helsinki revised in 2013 and actual French legislation, and the database was approved by our institutional review board. All patients included in the database signed the informed consent before undergoing prostate biopsy.

A total of 154 patients were retrospectively included in this analysis. All patients undergoing an MRI-guided prostate biopsy because of abnormally elevated PSA levels (>4 ng/ml) or/and abnormal digital rectal examination in our hospital were included. Two groups were formed: 78 participants in Group SOC received local anesthesia with 1% lidocaine periprostatic nerve block (PPNB) as recommended (18) and 75 patients in Group VR received an immersive video experience consisting of 360 experiences developed by the Healthy Mind company associated with PPNB on Oculus GO standalone Virtual Reality (VR) 64GB with a Bose QuietComfort 35 II headset. The Healthy-Mind VR® device offers virtual reality sessions based on computer-generated 3D images. These synthetic images are created in the smallest detail to unconsciously induce relaxation using principles of hypnotherapy, color therapy, cardiac coherence, and musicotherapy.

Demographics data, PSA levels, prostatic data (volume, finding on transrectal examination), characteristics of MRI-lesion (localization, PIARDS grade), patients' treatment (use of pain killers, anticoagulants, or antiplatelet therapy), and past biopsies were reported for all patients. One patient was excluded because the probe could not be introduced, and general anesthesia was necessary.

### 2.2. Analgesic techniques and biopsy

In Group SOC, the subjects were placed in the lithotomy position. An intrarectal instillation of an anesthetic gel was applied first. Then, the examiner realized the digital rectal examination and introduced the ultrasound (US) probe to practice the periprostatic nerve block. It took five minutes for the analgesia to have an effect and to do the ultrasound and MRI target mapping. The same steps were completed for Group VR and we also gave the patient glasses and a VR headset shortly after the positioning. The VR headset was provided by the Healthy Mind company in Paris. Subjects chose between three landscapes: snow world, forest walk, or zen garden. The glasses and the headset were removed at the end of the biopsy.

For each subject, a twelve-biopsy scheme combined with four biopsy cores per target was performed as recommended (10, 11). The procedure was done by two urologists who had more than two years of experience.

### 2.3. Pain score and follow-up

At the end of the procedure, the patient's pain was assessed by using the visual analog scale (VAS) in which 0 represents no pain and 10 represents the worst pain ever felt (19). For patients who needed Entonox association because of insurmountable pain, their VAS was considered as 10 to make an intention to treat analysis.

Then, the secretary called the patient within three days of the biopsy and asked them to quote the VAS on the third day. She also recorded if there were complications such as haematuria, urine retention, or fever. The patient was reviewed in consultation by the urologist for the diagnostic announcement 1 month after the biopsy. The pain felt during the biopsy was recorded again with a new VAS score. Patient satisfaction with the anesthesia technique was evaluated by asking the question “Are you satisfied with the current anesthesia method for prostate biopsy?” and afterward, “If you need to repeat the biopsy later, would you prefer general anesthesia?”. Their answers were recorded as “yes” or “no”.

### 2.4. Statistical analysis

Results were reported as mean and standard deviation (SD) for continuous variables and as percentages for categorical variables. Differences were assessed using the Mann–Whitney U test for continuous variables and the chi-square or Fisher exact tests for categorical variables.

The comparison of mean pain scores was stratified and performed according to the VR status (classified as group VR vs. group SOC) and assessed by Student tests. In the second step, naïve PB patients were selected and stratified according to the VR status (classified as group PB naive with VR vs. group PB naive with SOC) and assessed by Student tests.

Finally, the effect of VR adjunctive on survival was analyzed using a multivariate using logistic regression ANCOVA model that incorporated all covariates having *P* < 0.05 in univariate analysis. All statistical tests were 2-sided, and *p* < 0.05 was considered statistically significant. R software version 4.1.2 was used for all statistical analyses.

## 3. Results

There was no significant difference between the two groups in the baseline variables (Table 1). The mean age of participants was 66.95 years and the mean of the number of biopsy cores was 18.5. Only two patients, one in each group, had long-term painkiller treatment. In all, 50 patients had already had a prostate biopsy: 28 in Group SOC and 22 in Group VR (*p* = 0.35).

**Table 1:** Baseline and peribiopsy characteristics.

	All patients	Group A	Group B	*P*-value
	(*n* = 153)	PPNB	PPNB + VR	
		(*n* = 78)	(*n* = 75)	
Age	66.95 ± 7.4	66.6 ± 6.5	67.1 ± 8.4	0.6
PSA (ng/ml)	7.36 ± 3.5	7.2 ± 4	7.4 ± 3.0	0.7
Biopsy cores	18.47 ± 5	18.3 ± 6.1	18.7 ± 3.7	0.6
Prostatic volume (cc)	59.58 ± 26	62.8 ± 27.3	56.1 ± 24.9	0.1
Rectal touch				0.4
<T1c	118	62	56	
T2a-T2b	21	8	13	
>T2c	13	7	5	
Antiplatelet therapy	10	6	4	0.7
Painkiller habit	2	1	1	1
Previous biopsy	50	28	22	0.35
Gleason grade				0.9
No cancer	69	35	34	
Grade 6	43	21	22	
Grade 7	23	13	10	
Grade 8 and more	18	9	9	
D’Amico grade				0.9
No cancer	70	35	35	
Low risk	35	17	18	
Intermediate risk	39	21	18	
High risk	5	2	3	
MRI location				0.3
Apex	50	22	28	
Base	46	27	19	
Middle	51	27	24	
MRI side				0.3
Unilateral	92	50	42	
Bilateral	61	28	33	
				0.09
Anterior	10	8	2	
Posterior	142	69	73	
PIRADS				0.7
2	3	1	2	
3	16	6	10	
4	54	27	27	
5	79	43	36	
Painkillers after biopsy				0.8
Yes	19	9	10	
No	114	57	57	
Hematuria after biopsy				0.08
Yes	89	49	40	
No	48	19	29	
Urine retention after biopsy				1
Yes	9	4	5	
No	128	64	64	
Urinary tract infection after biopsy				0.7
Yes	7	4	3	
No	130	64	66	
Agree to return				0.6
Yes	122	59	63	
No	20	11	9	
GA preference				0.9
Yes	22	11	11	
No	119	58	61	

PPNB, periprostatic nerve bloc; VR, virtual reality; PSA, prostatic specific antigen; MRI, magnetic resonance imaging; PIRADS, prostate imaging reporting data system; GA, general anesthesia. Data are presented as mean ± SD.

Table 1 shows 84 patients with a positive biopsy, 43 and 41 patients, respectively, in Groups SOC and VR (*p* = 0.9); no other kind of cancer was found. Few adverse biopsy events such as hematuria or urinary retention were observed (Table 1). Four and three patients, respectively, in Groups SOC and VR developed a urinary tract infection, which led to hospitalization after the biopsy (*p* = 0.7). A total of 59 patients agreed to return for the same analgesia in Group SOC and 63 patients in Group VR (*p* = 0.6). Only 11 patients in each group would have preferred general anesthesia (*p* = 0.9).

There was no significant difference found between Group SOC and Group VR on the primary outcome. The mean pain score at day 0 was 3.4 (±2.5) and 2.9 (±2.3) for Groups SOC and VR (*p* = 0.2), respectively. The mean pain score on day 3 was 0.39 (±1.5) and 0.27 (±1.0) for Groups SOC and VR (*p* = 0.5), respectively (Table 2A).

**Table 2:** (A) Pain score in MRI-guided core biopsy in Group SOC (PPNB) and Group VR (PPNB and VR). (B) Pain score in MRI-guided core biopsy in subpopulation of naïve PB patients in Group of naïve with PPNB (SOC) and Group of naïve with PPNB and VR (VR).

(**A**)				
Pain score	All patients	Group SOC	Group VR	*P*-value
		PPNB	PPNB + VR	
	(*n *= 153)	(*n *= 78)	(*n *= 75)	
D0	3.18 ± 2.4	3.4 ± 2.5	2.9 ± 2.3	0.21
D3	0.33 ± 1	0.39 ± 1.5	0.27 ± 1.0	0.45
(**B**)				
Pain score	(*n *= 102)	Group SOC naïve	Group VR naïve	*P*-value
		PPNB	PPNB + VR	
		(*n *= 50)	(*n *= 52)	
D0	3.40 ± 2.4	3.8 ± 2.5	2.7 ± 2.3	0.012
D3	0.35 ± 1	0.45 ± 1.5	0.25 ± 1.0	0.024

When stratification on PB status (naïve or non-naïve) was used, the VR effect on pain score was slightly lower between non-naïve PB patients but remained highly significant between naïve-PB patients as compared to SOC vs. VR [3.8 (±2.5) vs. 2.7 (±2.3); *p* = 0.013)] at day 0. Similar results were found on day 3 for the analysis including naïve-PB patients with SOC vs. with VR [0.4 (±2.5) vs. 0.2 (±2.0); *p* = 0.023)] (Table 2B).

By multivariate analysis of the predictive factors of pain score at day 0 (Table 3), only one factor was found to decrease VAS, namely, MRI target base location [OR = −0.236, 95% CI (−0.440; −0.033), *p* = 0.023].

**Table 3:** Predictive factors of VAS pain score at Day 30 (*p* = 0.05).

Variable	Odd ratios	CI 95%	*P*-value
Age	−0.030	(−0.206; 0.147)	0.741
Volume prostate	−0.006	(−0.184; 0.172)	0.947
Hematuria			
No	−0.065	(−0.242; 0.112)	0.470
Yes	0	–	–
Urinary retention			
No	−0.0389	(−0.556; −0.221)	<0.0001
Yes	0	–	–
Urinary tract infection			
No	−0.047	(−0.218; 0.124)	0.587
Yes	0	–	–
Previous biopsy			
No	−0.047	(−0.220; 0.127)	0.596
Yes	0	–	–
Analgesia			
PPNB	0.113	(−0.058; 0.285)	0.193
PPNB + VR	0	–	–

Moreover, regarding pain score at day 30 (Table 3), only one side effect, namely, urinary retention was a predictive factor [OR = −0.0389, 95% CI (−0.556; −0.221), *p* < 0.0001]. Hematuria and urinary tract infection were not predictive factors. Finally, the analgesia technique (PPNB or PPNB and VR) did not affect the VAS pain score on day 30.

## 4. Discussion

The objective of this study was to evaluate the effect of VR as adjunctive in pain management in transrectal MRI-guided prostate biopsy in patients who underwent PB without general anesthesia in our urology department. For that purpose, we retrospectively analyzed the overall pain intensity reported in the Department database, a repertoire of patients admitted for care at Chalon Hospital (Chalon sur Soane, France). We also limited the heterogeneity of the population by analyzing the subgroup of patients, including only naïve PB patients enrolled between 2019 and 2021, who received local anesthesia with a 1% lidocaine periprostatic nerve block (BPNP).

We found in this population a more pronounced pain score reduction in naïve patients with PPNB associated with VR than in naïve patients with PPNB (Table 3). This study supports the hypothesis that VR associated with periprostatic nerve block is better than periprostatic nerve block alone for analgesia for patients undergoing transrectal MRI-guided biopsy.

Our study population is quite similar to other studies' populations. Indeed, mean age (14, 16, 17) and prostate volume (14, 20, 21) were equivalent. Moreover, few adverse events were observed: our complication rate was less than 5% as seen in the literature (22). Virtual reality should have the best tolerance compared to other pharmacological techniques. The tolerability of virtual reality was excellent in this study. No patients were concerned about vertigo, headache, nausea, or vomiting. However, the number of biopsy cores was clearly higher in our study: a mean of 18,5 cores was performed, whereas other studies did between 5,6 and 14 cores only (16, 20, 21).

A considerable amount of literature has been published on prostate biopsy analgesia. Regarding pain intensity, visual analog scales in literature were mostly lower than 4, which is consistent with our observation where the mean pain score was 3,18 (23, 24). Pain scores in studies over 4 mostly corresponded to placebo groups. According to our study design, visual analog scales were evaluated right after the biopsy procedure, in the same way as similar studies (12). This lack of hindsight explains why biopsy adverse events were not studied in the literature.

Several investigations have revealed that periprostatic plexus block in prostate biopsy, alone or in combination with intrarectal analgesia or sedation, is an effective method to reduce pain in comparison with placebo or intrarectal analgesia alone (12). But no studies proved the efficiency of adding VR to the PPNB. Nevertheless, recent evidence suggests that VR statistically reduces pain in medical procedures (25).

In reviewing the literature, no data was found on the assessment of VR as analgesia in prostate biopsy. Only one case report from 2005 about VR in urology was found and concerned transurethral microwave thermotherapy: association of PPNB, sublingual fentanyl, and lidocaine gel with VR revealed a pain decrease (8). A systematic review and meta-analysis of pediatric patients undergoing medical procedures showed pain and anxiety decreased with VR use (7). However, immersion quality seemed to be influenced by interaction with the virtual environment by means of changing position, changing orientation, perspective, and field of view. Indeed, differences between active and passive VR were described in a trial comparing passive and active VR scenarios. Tolerance of pain seemed to be better in active scenarios (26). Patients in our study had limited movements because of their lithotomy position and the precision our procedure required. This could probably explain our different results.

Moreover, pain score intensity was different from other VR investigations. A meta-analysis showed higher pain scores in control groups (analgesics alone) for the pain management of burnt patients undergoing dressing change or physical therapy (5). Their means of VAS were between 4.2 and 7.8, whereas our mean pain score right after the biopsy was 3.4 in our control group. Our results may be explained by the fact that VR is more efficient for higher pain scores.

Other components such as patients' age are important. Multivarious analysis showed that other variables such as age, prostate volume, and number of biopsy cores did not influence VAS scores as previously shown in the prostate biopsy literature (14, 16, 27). Indeed, most of these investigations with statistical differences between VR and control groups concerned populations younger than ours, such as trials about women in labor. VR would be effective for reducing pain in women in labor as compared to those receiving no intervention (4). However, the median age was 32.5 years (±3.6) for the control group and 31.6 years (±5.6) for the VR group, whereas our subjects were 59.55 years old and more. Studies on burn patients undergoing dressing change and physical therapy concerned young patients too (between 1.6 and 54 years old) (5). It seems possible that the difference in our results is linked to young generations being more receptive to VR. Indeed, a study about VR in orthopedics surgery also concerning an older population showed non-significant results (28).

The estimation of the effect of VR on pain management in the context of PPNB is subject to various sources of bias. The most problematic one is the bias of selection, i.e., the fact that patients who have already experienced prostate biopsy do not feel the same anxiety and pain as naïve patients and those patients have different demographic characteristics compared to others. Despite non-significant differences in overall patients, our investigation had strengths and showed the selection bias included in other studies, making it difficult to evaluate the effect of VR in this specific population. In this study, the analysis performed in naïve patients is consistent and robust. Our sample of the target population is comparable to many studies' populations, with few subjects and similar groups included in comparable studies. Including more subjects could have improved our study power.

Another limitation concerned the absence of pain score adjustment on biopsy steps. Indeed, pain scores may be higher during probe introduction than during anesthesia injection or core retrieval (13). In our analysis, a global pain score was evaluated for the whole procedure because we did not want to extract subjects from their immersive condition. Furthermore, this biopsy procedure cannot be made without a probe introduction.

In addition to this, the main study limitation is the lack of anxiety data. Several studies showed VR reduced fear and anxiety during dental procedures (6, 29). Further studies, which take these variables in prostate biopsy into account, will need to be undertaken.

This study supports the hypothesis that VR associated with periprostatic nerve block is better than periprostatic nerve block alone as analgesia for naïve PB patients undergoing transrectal MRI-guided biopsy. A VR treatment benefit may potentially exist in patients with previous experience of PB in terms of reduction of anxiety and increasing comfort. Further studies will be needed to evaluate whether this VR effect is specific to naïve PB patients or also exists in other biopsies procedures.

## Data Availability

The original contributions presented in the study are included in the article/Supplementary Material, further inquiries can be directed to the corresponding author.
